# Enhanced skills in global health and health equity: Guidelines for curriculum development

**Published:** 2017-04-20

**Authors:** Russell Dawe, Andrea Pike, Monica Kidd, Praseedha Janakiram, Eileen Nicolle, Jill Allison

**Affiliations:** 1 Memorial University of Newfoundland, Newfoundland and Labrador, Canada; 2 Women’s College Hospital, University of Toronto, Ontario, Canada; 3 St. Michael’s Hospital, University of Toronto, Ontario, Canada

## Abstract

**Introduction:**

Global health addresses health inequities in the care of underserved populations, both domestic and international. Given that health systems with a strong primary care foundation are the most equitable, effective and efficient, family medicine is uniquely positioned to engage in global health. However, there are no nationally recognized standards in Canada for postgraduate family medicine training in global health.

**Objective:**

To generate consensus on the essential components of a Global Health/Health Equity Enhanced Skills Program in family medicine.

**Methods:**

A panel comprised of 34 experts in global health education and practice completed three rounds of a Delphi small group process.

**Results:**

Consensus (defined as ≥ 75% agreement) was achieved on program length (12 months), inclusion of both domestic and international components, importance of mentorship, methods of learner assessment (in-training evaluation report, portfolio), four program objectives (advocacy, sustainability, social justice, and an inclusive view of global health), importance of core content, and six specific core topics (social determinants of health, principles and ethics of health equity/global health, cultural humility and competency, pre and post-departure training, health systems, policy, and advocacy for change, and community engagement).

**Conclusion:**

Panellists agreed on a number of program components forming the initial foundation for an evidence-informed, competency-based Global Health/Health Equity Enhanced Skills Program in family medicine.

## Introduction

Global health addresses health inequities in the care of underserved populations, both domestic and international.^1^ Training in this field is increasingly popular among medical postgraduates and is associated with increased recruitment to medical training programs.^2^–^7^ Among medical learners global health training is also associated with increased clinical competency and resource management.^8^–^10^

Given that health systems with a strong foundation in primary care are the most equitable, effective and efficient, family medicine is uniquely positioned to engage in global health.^11^,^12^ However, there are no nationally recognized standards in Canada for postgraduate family medicine training in global health and no evidence-based guidelines for designing program curriculum. There are currently five universities in Canada with a family medicine enhanced skills program (i.e., an additional year of residency training) in global health. Some of these programs have a more self-directed focus on curriculum, while others have a structured core content. Some of these programs have defined competencies for their learners, whereas others do not.^13^ In creating a new postgraduate training program at Memorial University for family physicians in the care of underserved populations we consulted medical educators with experience in primary care and global health (see Table 1) regarding approaches to global health enhanced skills education. Our aim was to establish what, if any, program features, content topics and methods of teaching and assessment should characterize all global health enhanced skills programs (GH ESPs) for family medicine residents.

The benefit of attempting to standardize global health education is a controversial topic. Some have argued that it is meaningless to set general competencies for work that might take place in vastly different contexts.^3^ However, several authors have suggested that there are aspects of global health that may be transferable between various contexts, and that these need to be identified for the education and assessment of global health learners.^14^–^16^ Postgraduate medical education today places a strong emphasis on achievable competencies,^14^,^17^,^18^ and any new program must develop curriculum according to clear objectives and outcomes in order for any meaningful evaluation (including national accreditation) to occur.^2^,^19^ This study aims to establish the broadly-applicable topics and their associated teaching methods that all family medicine GH ESPs should include (hereafter referred to as “core” material), as opposed to that material which may only be relevant to some programs and contexts. Non-core material may include topics that are appropriate to some GH ESPs but should not be required of all such programs. Identifying core themes of GH ESPs could contribute to the development of global health education and assessment while upholding that necessary flexibility unique to each program.

Much of the current literature on global health postgraduate medical education comes from the United States. The growth and activity of national bodies across Canada, such as the College of Family Physicians’ Besrour Centre and Global Health Committee, demonstrate increasing commitment to global health among Canadian family doctors, and the need for an increased Canadian contribution to this medical education literature.

### Research question

What do expert medical educators believe to be the essential components of a Global Health and Health Equity Enhanced Skills Program (GH ESP) in family medicine?

### Objective

Our objective was to use the Delphi method with a panel of Canadian experts in global health, medical education, and primary care to generate consensus on what core content, as well as best practices for delivery and assessment, should characterize all GH ESPs for family medicine residents.

## Methods

A local committee was formed at Memorial University of Newfoundland (MUN) to develop an enhanced skills program for family medicine residents focused on developing the skills needed to address the health equity challenges of underserved populations in various domestic and international contexts. A literature review and environmental scan of global health medical education was carried out for the committee by MUN’s Medical Education Scholarship Centre. Details regarding the literature review can be found in the supplementary information for this article (eSuppl 1). Using the results of this review and environmental scan, the committee developed a survey (available from the corresponding author upon request) meant to assess the most important elements of a family medicine GH ESP. This project was reviewed and approved by Newfoundland and Labrador’s Health Research Ethics Board (HREB reference #2016.027).

### Design

To develop an agreed-upon set of elements to include in family medicine GH ESPs, we administered the survey using the Delphi technique (a method of group communication designed to allow a panel of experts to reach a convergence of opinion on an issue related to their expertise).^20^ Measuring convergence of opinion allowed us to distil their views in order to find the program elements our experts believed should characterize family medicine GH ESPs and also highlighted the areas on which our experts could not agree. In the absence of nationally recognized standards in Canada for postgraduate family medicine training in global health and evidence-based guidelines for designing program curriculum, this method is a suitable starting point.^21^

A review of consensus measurement in Delphi studies across multiple disciplines revealed that there is not yet a generally accepted standard for the measurement of consensus in Delphi studies. In fact, this review highlighted 15 different methods of measuring consensus.^22^ For the purposes of this study, we selected a simple but effective method of measuring consensus: a predefined level of agreement.^22^ Specifically, we used a predefined and moderate estimate of consensus: selection of a response by a supermajority of at least 75% of the panellists (as used by Fox et al.) for dichotomous, Likert-type, and multiple response questions.^21^ Choosing a moderate level of agreement *a priori* allowed us to ensure that our results represented the views of a large majority of our expert panel and protected us from confirmation bias. For questions with other response options we report the median and interquartile range.

### Expert panel

In February 2016, we recruited Canadian medical educators and practitioners with global health experience to form an expert panel. To be eligible for inclusion, experts were required to be equipped with experience in: domestic (Canadian) and international (low and middle income countries) health care of underserved populations; medical education (especially but not exclusively in the training of family medicine residents and/or current and former family medicine GH ESP residents); and primary care (predominantly family medicine, making allowance for a limited number of nominees from other relevant fields such as community health, given their experience in GH and medical education and the significant overlap between these fields). We used snowball sampling to form a pool of potential candidates for the panel starting with the personal contacts of the research team, each of whom were then asked to nominate additional candidates. Demographic information for the panel of experts formed for this study is highlighted in Table 1 of the results section.

### Survey development

Guided by information gathered from the literature review and environmental scan, the research team developed a survey consisting of both fixed response and open-ended survey items. These items queried program features (e.g., length and objectives of a health equity/global health program), focus (domestic and/or international), content (learning topics and methods of instruction), and learner assessment. The resulting effort was reviewed and approved by the larger Care of Underserved Populations Program Development Committee. Next, the directors of the five Canadian GH ESPs reviewed the draft survey and provided feedback. Their minor changes were incorporated and the first iteration of the survey was formed. We used responses on these items as well as feedback provided between rounds to determine new questions and/or response options for the next iteration of the survey.

### Study protocol

**Figure 1:**
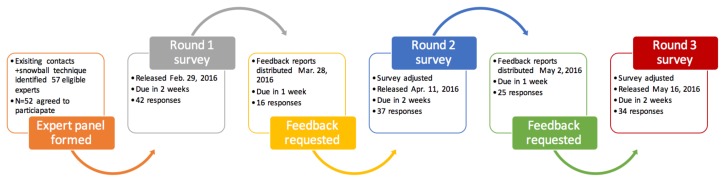
Process Diagram of Study Protocol

We administered the Delphi survey in three rounds using Fluid Surveys online survey software. We also provided participants with personalized reports of study results between rounds and requested their feedback in order to revise the survey for the next administration. See Figure 1 for a process diagram highlighting study protocol.

For each round of the survey, participants were given ten business days to respond; non-responders were emailed reminders on days six and nine. After the survey deadline, the team analyzed results within one week and prepared and delivered individualized reports to participants. These reports included the participant’s individual response as well as the aggregate group response for comparison. Participants were then given one week to reflect on their choices and return their feedback reports to the research team for review. All participants were given the option to provide feedback, but feedback was not required to move on to the next round of the survey and was not provided by all participants in each round. In round one, 16 of 42 participants provided feedback, in round two, 25 of 37 provided feedback, all of which was reviewed by the authors RD and AP. The perspectives of those who provided feedback were judged (by RD and AP) to be generally representative of the panel as a whole. The feedback informed the next iteration of the survey and was included in the results only when a respondent specifically requested to change their original response. Feedback on the final round was not requested. Responses were reviewed by RD and AP; they felt that responses were clear and that no unique or new information would be yielded from administering an additional iteration of the survey.

The second iteration of the survey was adjusted significantly to account for findings from the first round. Once a consensus was obtained, the relevant question was dropped from further iterations of the survey. One week after receiving feedback reports, the second iteration of the survey was distributed. The third and final iteration of the survey included only four questions on the remaining unresolved issues.

In order to protect against potential investigator bias, the research team was unable to link survey responses with participant identities. MUN’s Primary Healthcare Research Unit (PHRU) implemented the survey and managed the data.

### Outcome measures

We grouped our study outcomes into four major categories: program features, program focus, program content, and learner assessment. These outcomes were measured using various items from the three iterations of the survey used in our Delphi exercise (see Table 2 in Appendix).

### Data analysis

We computed descriptive statistics to analyze quantitative data from fixed response option questions using IBM SPSS Statistics 22. Qualitative data from open-ended questions were reviewed and coded for emerging themes by two authors (RD and AP). Results were then circulated to other members of the research team for their review and approval.

## Results

Fifty-seven eligible experts were identified and invited to join our panel of experts; 52 individuals (91%) agreed. Forty-two of those 52 experts (81%) returned a completed survey in round one. Only those who completed the previous round of the survey were asked to complete the remaining iterations. Thirty-seven of 42 round one participants (81%) returned a completed survey in round two, and 34 of 37 round two participants (92%) returned a completed survey in round three. Thirty-four of 57 invited participants (60%) completed all three rounds of the survey. Please see Table 1 for a summary of the panel’s demographic information.

### Program features

Frequencies of responses for survey items addressing program features are presented in Table 2 (Appendix).

#### Optimal Program Length (N=42)

The findings were clear that a 12-month program is preferable to 6 months. 85.7% of panellists (n=36) reported that 12 months is the optimal length for a family medicine enhanced skills program in global health.

#### Mentorship (N=34)

Mentorship had a clear and unequivocal place in family medicine GH ESPs, as 100% of panellists (n=34) reported that all residents in a family medicine GH ESP should work with a mentor while they are completing the program. A supermajority of participants agreed that a mentor should model health equity in their own careers (88.2%, n=30), guide the reflective learning of the resident as they progress through the program (88.2%, n=30), guide residents in program and placement choices (88.2% n=30), and provide academic support to learners by directing them to additional literature or contacts as relevant to their learning needs (82.4%, n=28). Free-text comments support these findings.

Mentors are important resource to act as sounding boards, reflective opportunities and provide guidance to help frame goals and objectives, can assist in facilitating connections for placements.

I think experienced mentorship should be a pillar of any successful program. The role of the mentor is to serve as a resource for links/contacts, to actively demonstrate how a career in global health can be balanced with work/family life in Canada, and to continually challenge the student to question their stereotypes and assumptions.

#### Research (N=34)

Respondents remained divided on the issue of research as 50% of panellists (n=17) reported that participating in some form of research activity should be a mandatory component of a GH ESP and half did not. However, 91.2% (n=31) felt that family medicine residents enrolled in a GH ESP should learn how ethics applies in the context of research with international or vulnerable populations, and 76.5% (n=26) felt that residents in this type of program should learn how to use research for advocacy. In addition, free-text comments we received from the second iteration of the survey indicate that program support for research and scholarly activity by interested learners is encouraged, and such scholarly activity should be undertaken in an accountable and collaborative manner over an adequate period of time. In addition, some panellists suggested that scholarly projects could be longitudinal, engaging the community with support from GH ESP faculty, with periodic contributions from various GH ESP residents over the course of several years.

I think that research opportunities should be provided and strongly supported for those students with an interest in pursuing it.

I would suggest that a project (scholarly or otherwise) that is initiated with a community/community agency/community organization where the power for the determination of what the project will be, how it will be conducted, etc. is a shared by the community and the resident is important. However, it needs to be clear that the process is more important than the product. Given that this is likely to be only a year long program, engaging with community for this sort of activity can take much longer than a year so it would be important that the resident not be solely focussed on a completed project but rather the process of engagement.

#### Program objectives (N=37)

A supermajority of participants believe that a GH ESP should foster the development of advocacy skills (78.4%, n=29), emphasize the importance of sustainability in global health activities (89.2%, n=33), highlight the essential role of social justice in health (86.5%, n=32), and cultivate a view of global health that includes domestic and international populations (89.2%, n=33).

### Program focus

Frequencies for survey items addressing program focus are presented in Table 2 (Appendix). Results (median and the interquartile range (IQR) distribution of scores) for items assessing the proportion of time that should be devoted to particular aspects of the program can be found in Table 3.

#### Single versus dual focus (N=37)

A supermajority of respondents, 75.7% (n=28) reported that a family medicine GH ESP should address both domestic and international health, spending a median of 50% of their time (IQR = 40, 63) on domestic issues. Further, among the nine panellists who reported that a single focus was *reasonable*, all but one panellist reported that a dual focus is *preferable*. Similarly, participants reported that residents in a family medicine GH ESP should spend a median of 50% of their time on rural health issues (IQR = 50, 50).

Several respondents further explained that learners benefit from both domestic and international practica as well as a blend of both rural and urban training that informs their future practice settings and academic contributions. Furthermore, governments funding such programs often desire a local return on their investment through domestic service.

I believe that a program should allow trainees to experience both domestic and international health, as a good understanding of both, will serve to enhance the future work of the trainee whether they choose [to practice] domestically or internationally.

Ideally, there is a balance between the two and through reflection the residents can see similarities and differences. I think this strengthens the experience and ability to transfer skills and approaches to new contexts.

### Program content

Results for survey items addressing program content are presented in Table 2 (Appendix). Results (median and the IQR distribution of scores) for items assessing the proportion of time that should be devoted to particular aspects of the program can be found in Table 3.

#### Importance of core content (N=37)

A supermajority of experts (81%, n=30) felt that core content should be included in a family medicine GH ESP and that residents should spend a median of 50% of their time focused on core content (IQR = 38, 65).

Respondents commented that having consistency across programs will consolidate the scope and language that particularizes the field of health equity and global health. This consistency also provides residents with clarity regarding the program’s expectations, and enables a focused program evaluation that facilitates ongoing revisions to core components of curriculum from year to year.

Having a core content ensures a certain degree of direction and quality control for the program. It helps shape the mandate of the program and its faculty.

Global health is a lens. To me, clinical skill acquisition is less crucial (and should not be core requirements) during a global health residency than understanding the ethical, political and social justice considerations of global health (which should be core components).

#### Core learning topics (N=37)

Panellists reached consensus on six of the 12 learning topics presented. A supermajority agreed that the following topics should be included as part of a core curriculum in a GH ESP:

1. Social determinants of health (100%, n=37)
2. Principals and ethics of health equity/global health (100%, n=37)
3. Cultural humility and competency (94.6% n=35)
4. Pre and post-departure training (86.5%, n=32)
5. Health systems, policy, and advocacy for change (81.1%, n=30)
6. Community engagement (75.7%, n=28)

#### Non-core learning topics (N=42)

Panellists also agreed that the following topics should *not* be required of all learners as core curriculum:

1. Procedures (97.6%, n=41)
2. Traveller’s medicine (83.3%, n=35)
3. Humanitarian response (88.1%, n=37)
4. Outbreak management/epidemiology (81.1%, n=34)

Although they did not reach supermajority, there were a number of specific areas of medicine (e.g., global burden of non-communicable disease) and particular population groups (e.g., Indigenous populations), which are common in GH ESPs and which a small majority of panellists did rate as core content (see Table 2 in Appendix). In essence, most panellists felt that some medicine topics or population groups should be core, but they did not agree on which ones. The fact that none of these topics garnered a supermajority as core suggests that, while they may be of great value to a family medicine GH ESP, it is reasonable for a program to take a resource-based approach to which specific populations and areas of medicine they include in their program.

#### Learning formats (N=37)

Data analysis revealed a median of 65% (IQR 60, 75) of the learning in a GH ESP should occur through fieldwork, with the balance of time spent on study. We defined fieldwork as “direct patient care, resident-delivered clinical teaching, community engagement, and health systems/policy experience.” Study included “independent or group work such as assigned readings, online modules/courses, short courses away, and small group learning.” Our results support an emphasis on learning through fieldwork.

### Learner Assessment (N=37)

Frequencies for responses to the survey items about learner assessment are presented in Table 2 (Appendix).

Panellists reached consensus on the relative importance of four general categories of learning assessment. The individualized portfolio (consisting of a collection of essays written, experiences reflected upon, literature reviewed, certificates earned, etc.) was ranked as the first or second choice of assessment by 91.9% of panellists (n=34). In-training evaluation reports (ITER) were ranked as the first or second choice of assessment by 75.7% of panellists (n=28). Participation was ranked as the third or fourth choice by 86.5% of panellists (n=32). Reflection essay was ranked as the third or fourth choice of assessment by 81.1% of panellists (n=30).

## Discussion

The program features identified in our findings are generally consistent with current literature and practice in GH ESP education. Family medicine enhanced skills programs in Canada are accredited by the College of Family Physicians of Canada at a length of twelve months or less.^23^ Global health fellowships of all specialties throughout the United States are usually 19 – 24 months’ duration, many of which are integrated throughout residency.^24^ Given this length, it is understandable that the full twelve months available to a GH ESP in Canada would be desirable. A longer program presumably leads to a better opportunity to build meaningful relationships with marginalized or vulnerable patients and communities that have been engaged by their GH ESP.

Mentorship is fundamental to teaching global health, particularly its attitudes and values, to family medicine residents.^18^ Our findings regarding the role of a mentor in a GH ESP are consistent with previous global health medical education literature in undergraduate and postgraduate contexts. Mentors should: help residents process learning^13^ and tailor the program to their interests,^25^,^26^ be a role model of global health in their own career^13^ and a resource for the resident’s learning,^12^,^18^,^25^,^27^ help residents navigate program’s objectives^25^ and provide career counselling.^25^,^26^ Mentorship may be a natural result of working cross-culturally, requiring a mentor with experience in both cultures to interpret the significance of the resident’s experience, clinically, interpersonally, or otherwise.

Many global health programs have sought the synergy of combining mentorship with research in a single relationship between resident and faculty around a research project.^25^ Research in global health bears particular ethical and methodological considerations, which emphasize the need for mentorship. Ethics include reciprocity and relevance to host community^25^,^26^ and methods include community-based or participatory action approaches,^28^ to which a resident may not be sensitive.^3^ Unfortunately, research is lacking on international and community hosts’ perspective regarding collaborating with global health residents and faculty in research.^28^ Additionally, for every task (e.g., research) required of a busy resident, some other learning opportunity is foregone.^29^ The divide in our panellists’ perspectives on mandatory research projects in a GH ESP may reflect their awareness of these challenges in global health research. However, combining research with appropriate mentorship may mitigate these risks, and a resident may better engage in global health research by contributing to a part of a longitudinal project overseen by a faculty mentor.^28^ Participating as one member of a research team may provide a resident a lighter load of research, thus partially addressing the challenge of competing priorities for busy learners.^29^

The value of both domestic and international experience during global health training is well described in the literature.^4^,^16^,^18^,^25^,^26^,^28^,^30^,^31^ Global health fellows throughout the United States commonly work at home and abroad.^24^

The core content topics identified in our study by a supermajority (see Table 2 in Appendix) are all well represented in recent global health literature.^4^,^25^,^31^,^32^ There is, however, a notable absence among our findings of any specific areas of medicine or particular population groups which should be addressed by all GH ESPs. This contrasts with a number of previous publications and programs which have listed the following among their recommended competencies: maternal/newborn care, traveller’s medicine, communicable/tropical diseases, mental health, immigrant/refugee care, chronic disease and more.^4^,^31^,^33^ This divergence from previous literature returns to our original question of what minimum curricular content should be met by all family medicine GH ESPs in Canada. There are several reasons that may have led to this departure from previous recommendations:

First, family medicine is inclusive of all human demographics, and is the appropriate first line of care for most medical and social issues, at a wide range of acuity. Specialists in global health are more likely to operate in a context where specific clinical issues must be included. For example, where a paediatrician practices global health, it may be reasonable to make newborn care a required topic.

Second, enhanced skill programs will take learners who have already achieved the clinical competencies required for domestic practice, and relatively few graduates of fellowship programs in GH actually go on to practice internationally.^27^,^32^

Third, our core topics are not intended to comprise a comprehensive list of competencies for a GH ESP, but to establish a common foundation upon which additional skills and knowledge may be added. Our findings suggest that the transferable skills and knowledge necessary for all GH ESPs belong to the communicator, collaborator, scholar, professional, leader, and health advocate CanMEDS competency roles.^17^ However, no medical education program can ignore the medical expert role entirely.^17^,^18^ In fact, a supermajority of all respondents (76%, n=32; data not shown) identified at least one medical area or special population as core material. This would suggest that medicine is required, but which areas to teach can be flexible.

One possible exception to this flexibility is the social determinants of health, agreed upon by 100% of respondents (n=37) as a core topic. The social determinants of health could be included under the medical expert,^31^ but they extend beyond clinical medicine to address the structural and social factors that impact the health of communities. While all GH ESPs should address this core topic thoroughly, institutions seeking to establish a competency-based program for a GH ESP will need to exceed this and the rest of the minimum common ground found by our study in order to sufficiently include the role of medical expert.^13^,^25^

Teaching postgraduate global health commonly includes a mix of methods and experiences, such as clinical experience, curriculum (written, online, classroom, etc.), and research.^24^,^27^ Methods of assessment vary widely. Family medicine residency programs most often evaluate their residents’ global health activities by having a supervising physician complete their evaluation,^27^ such as an In-Training Evaluation Report (ITER). Scholarly presentations, self-assessment, reflections, and mentor’s assessment were each used in 14.1 – 15.8% of programs.^27^ Our study identified the ITER and portfolio as the top two forms of assessment for residents in a GH ESP.

Portfolios provide a cumulative and flexible mode of competency-based assessment,^34^ which is well-suited to the motivated and experienced learners who enroll in enhanced skills programs. Portfolios have a number of qualities that make them uniquely suited to assessment in global health. First, portfolios can assess a broad scope of competency domains, including attitudes, values, communication skills, and professionalism, which are fundamental to global health but often difficult to assess.^35^,^36^

Portfolios can reflect a resident’s interpretation of and response to difficult experiences, which is important when assessing cultural humility. Second, portfolios allow a resident to gather evidence of their transformative learning and competency over a period of time, including input from multiple media and sources.^35^–^37^ This flexibility accommodates changes to placements and their preceptors, enabling feedback from community partners and other colleagues, which may not be adequately captured by an ITER. Finally, portfolios are most effectively used in the context of a learner-mentor relationship,^34^,^36^,^37^ which is a key component of teaching and assessment in global health education.^27^

### Limitations

Expert consensus provides a low level of evidence. Methods to establish and quantify outcomes in the field of health equity and global health have yet to be defined and validated. We have sought to establish initial guidelines in the hope that it will lead to evidence-based educational programming, and subsequent rigorous study to revise these initial guidelines.

This study’s panellists and, therefore findings, represent a small sample of a limited perspective. There is a lack of political neutrality in our findings, and the questions of who contributes to a set of standards and who benefits from their creation should not be overlooked.^38^ This conversation would therefore benefit from input provided by current residents and graduating PGY2 family medicine residents, inter-professional colleagues, policy makers, NGO’s, and especially international partners and the underserved populations for whom we aim to provide care.

### Conclusion

In summary, there is a wide range of opinion among academic family physicians with expertise in health equity and global health as to what should be included in a GH ESP. While there is agreement for a few guidelines for such a program’s features, focus, content, and learner assessment, so much of what is experienced in a GH ESP remains to be defined, such as which areas of medicine to teach, and which particular underserved populations to engage. This indicates that a degree of flexibility regarding program content should be expected. Individual programs should apply these findings in accordance with their own contexts and then evaluate their efficacy over time to see which elements hold up after implementation.

## Supplementary Information



## Figures and Tables

**Table 1:** Panel Demographics (N=42)

Category		n (%)

Gender	Male	19 (45.2)
	Female	23 (54.8)

Region	Atlantic Region (NL, NS, NB, PEI)	2 (4.8)
	Central Region (QB, ON)	22 (52.4)
	Prairie Region (AB, MB, SK)	12 (28.6)
	Pacific Region (BC)	5 (11.9)
	Northern Region (NWT, YK, NU)	1 (2.4%)

Focus of Global Health Work	Domestic	23 (54.8)
	International	19 (45.2)

Global Health Experience	Learner	14 (33.3)
	Educator	21 (50)
	Other	7 (16.7)

**Table 3:** Proportion of time devoted to aspects of a family medicine global health/health equity enhanced skills program.

Outcome	Survey Item	Median	IQR
Program focus	For programs with a dual focus, what proportion of time should be spent on domestic versus international health	50% on domestic	40, 62.5
	What proportion of time should be spent on rural versus urban issues	50% on rural	50, 50
Program content	Overall what proportion of time should be spent on core content	53% on core content	38, 65
	Overall what should be the balance of fieldwork versus study	65% on fieldwork	60, 75

## Appendix

**Table 2:** Frequencies for responses on all survey items including both those that did and did not reach consensus

Survey Item			n (%)
**Program Features**			
**Program Length (N=42)**			
Optimal length of global health family medicine enhanced skills program	12 months		36 (85.7)
	Other		4 (9.5)
	6 months		2 (4.8)
**Mentorship (N=34)**			
Should all residents work with a mentor	Yes		34 (100)
	No		0
Role of mentor	Help residents process learning		30 (88.2)
	Help residents tailor program to their interests		30 (88.2)
	Be a role model of global health in their own career		30 (88.2)
	Resource for the resident’s learning		28 (82.4)
	Help residents navigate program’s objectives		21 (61.8)
	Provide career counselling		21 (61.8)
**Research (N=34)**			
Should participating in research be mandatory program feature	Yes		17 (50)
	No		17 (50)
Residents should:	Learn how ethics applies in context of research with vulnerable populations		31 (91.2)
	Learn how to use research for advocacy		26 (76.5)
	Learn research methods for community-based work		22 (64.7)
	Learn how to evaluate programs		21 (61.8)
	Contribute to an ongoing project (where possible)		20 (58.8)
	Work with community to develop sustainable project		19 (55.9)
	None of the above		2 (5.9)
**Program Objectives (N=37)**			
A family medicine global health program should foster:	An understanding of the importance of sustainability in global health activities		33 (89.2)
	An inclusive view of global health that includes domestic and international populations		33 (89.2)
	An understanding of the role of social justice in health		32 (86.5)
	Development of advocacy skills		29 (78.4)
	An understanding of key stakeholder roles and health systems		27 (73.0)
	An understanding of the importance of reciprocal relationships		25 (67.6)
**Program Focus**			
**International and/or Domestic Focus (N=37)**			
Is it reasonable to focus solely on international or domestic health	No		28 (75.7)
	Yes		9 (24.3)
Is a single focus preferable to a dual focus (N=9)	No		8 (88.9)
	Yes		1 (11.1)
**Program Content**			
**Core Content (N=37)**			
How important is the inclusion of some core content	Important – very important		30 (81.1)
	Not at all important – somewhat important		7 (18.9)
Should these topics be core or non-core	Social Determinants of Health	Core	37 (100)
		Non-core	0
	Principles and Ethics of Global Health	Core	37 (100)
		Non-core	0
	Procedures (N=42)	Core	1 (2.4)
		Non-core	41 (97.6)
	Humanitarian response (N=42)	Core	5 (11.9)
		Non-core	37 (88.1)
	Cultural Humility and Competency	Core	35 (94.6)
		Non-core	2 (5.4)
	Pre and Post-departure Training	Core	32 (86.5)
		Non-Core	5 (13.5)
	Traveller’s medicine (N=42)	Core	7 (16.7)
		Non-core	35 (83.3)
	Health Systems, Policy, Advocacy for Change	Core	30 (81.1)
		Non-core	7 (18.9)
	Outbreak management/epidemiology (N=42)	Core	8 (19.0)
		Non-core	34 (81.0)
	Community Engagement	Core	28 (75.7)
		Non-core	9 (24.3)
	Mental Health and Addictions	Core	10 (27.0)
		Non-core	27 (73.0)
	Inner City Health	Core	11 (29.7)
		Non-core	26 (70.3)
	Refugee/Immigrant Care	Core	16 (43.2)
		Non-core	21 (56.8)
	Global Burden of Disease	Core	20 (54.1)
		Non-core	17 (45.9)
	Maternal and Child Health	Core	17 (45.9)
		Non-core	20 (54.1)
	Indigenous Health	Core	19 (51.4)
		Non-core	18 (48.6)
	Infectious Disease	Core	18 (48.6)
		Non-core	19 (51.4)
**Learner Assessment**			
**Forms of Assessment (N=37)**			
Rank these forms of assessment in order of importance:	Individualized Portfolio ranked 1^st^ or 2^nd^ choice		34 (91.9)
	ITER ranked 1^st^ or 2^nd^ choice		28 (75.7)
	Participation ranked 3^rd^ or 4^th^ choice		32 (86.5)
	Reflection essay ranked 3^rd^ or 4^th^ choice		30 (81.1)
