# Supplementary material for: Enhanced skills in global health and health equity: Guidelines for curriculum development
Source: Can Med Educ J. 2017 Apr 20;8(2):e48–60. (PMC5669293)
Supplement: Supplementary file 1 [file CMEJ-08-48-eSuppl_1.pdf]

eSuppl1. Literature Review

A literature review and environmental scan surrounding global health medical education was carried out for the committee by Memorial's Medical Education Scholarship Centre. We searched the PubMed database in 2016 for Canadian and international articles published in the preceding 5 year period using the following search terms: ("Primary Health Care"[Mesh] OR "General Practice"[Mesh] OR "Physicians, Family"[Mesh]) AND ("global health"[MeSH Terms] OR "global health"[All Fields] OR "health equity"[All Fields]) AND ("Internship and Residency"[Mesh] OR "Education, Medical"[Mesh:NoExp] OR "Education, Medical, Graduate"[Mesh] OR "Program Develpment"[Mesh]). Similar terms were used to search Embase, Scopus, and ERIC. A total of 46 articles were identified and reviewed.

In addition to a search of peer-reviewed literature, an environmental scan was conducted to identify Canadian family medicine programs offering a family medicine GH ESP. This began with an e-mail to all Canadian family medicine program directors requesting contact information for a faculty lead for global health within their department. We identified family medicine GH ESPs already in operation at 5 universities: Queen's University, University of British Columbia, University of Calgary, University of Ottawa, and University of Toronto. Follow up conversation with the program directors of each of those GH ESPs identified no further family medicine GH ESPs elsewhere in Canada.
